# Complementary Remedies

**Published:** 1896-06

**Authors:** C. L. Olds

**Affiliations:** Philadelphia, Pa.


### COMPLEMENTARY REMEDIES.

C. L. Olds, M. D., Philadelphia, Pa.

Very often in the treatment of a patient, no matter whether the disease be acute or chronic, we find that after a longer or shorter period of time the remedy that was indicated in the beginning of the treatment no longer benefits the patient. We say that the remedy has "run out," and that another remedy must be selected. If, after the administration of this remedy the patient progresses toward health, the second remedy, because it completes the work of the first in a greater or less degree, is called a complement of that remedy. For example, we may find that a patient improves under Sulph. in its various potencies for a long period, but there comes a time when Sulph. no longer acts beneficially, and on restudying the case we find Calc. to be the remedy corresponding to the state of the patient; then, if there be improvement again, we say that in this case Calc. was the complement of Sulph. But there may come a time when Calc. is no longer of use to the patient, and on again studying the symptoms we are very apt to find Lyc. the remedy, if it be a chronic complaint. Here Lyc. is the complement of Calc.

It has been found by experience and a knowledge of the action of certain remedies that when one remedy is given and benefits the patient, but does not restore to health, that a certain other remedy will in all probability be the one to take the case up and carry it along to or toward health, as in the case of the Sulph. and Calc., cited above.

The following list of complementary remedies has been gathered from our works on materia medica, current literature, and the experience of several well-known physicians; some notes of the late Dr. Adolph Lippe, also forming a part of the work.

| | COMPLEMENTS. |
|---|---|
| Abrotanum. | Bry., Kali-bichr., Lyc. |
| Acetic-acid. | Chin. |
| Aconite. | Arn., Coff., Millef., Spong., Sulph. |
| Æthusa. | Calc. |
| Allium sativa. | Ars. |
| Aloes. | Sulph. |
| Alumina. | Bry., Ferr. |
| Antimonium-crudum. | Scill. |
| Apis. | Natr-mur., Hell. |
| Argentum-nitricum. | Natr-mur. |
| Arnica. | Acon., Psor., Rhus, Sulph-ac. |
| Arsenicum. | All-sat., Carb-veg., Lach., Natr-Sulph., Phos., Sulph., Thuj. |
| Baryta-carb. | Ant-t. |
| Belladonna. | Borax, Calc., Natr-mur. |
| Bryonia. | Abrot., Alumn., Kali-carb., Rhus, Sep., Sulph. |
| Bufo. | Salamandra. |
| Caladium. | Nitr-ac. |
| Calcarea-carb. | Lyc. |
| Calcarea-phos. | Ruta, Sulph., Zinc. |
| Calendula. | Hep. |
| Carbo-an. | Calc-phos. |
| Carbo-veg. | Ars., Kali-carb., Lach., Phos. |
| Causticum. | Petros. |

| | |
|---|---|
| Cepa. | Phos., Puls., Sars., Thuj. |
| Chamomilla. | Bell., Calc., Magn-c. |
| China. | Ars., Calc-phos., Ferr. |
| Cina. | Calc., Sulph. |
| Colocynthis. | Merc., Staph. |
| Corallium-rubrum. | Sulph. |
| Crotalus. | Carb-veg. |
| Cuprum. | Ars., Calc., Iod. |
| Drosera. | Carb-veg., Nux. |
| Dulcamera. | Alum., Bar-c. |
| Ferrum. | Alum., Ars., Chin., Ham. |
| Fluoric-acid. | Sil. |
| Graphites. | Ars., Caust., Ferr., Hep., Lyc. |
| Helleborus. | Zinc. |
| Hepar. | Sil. |
| Ignatia. | Natr-mur. |
| Iodine. | Bad., Lyc. |
| Ipecacuanha. | Cupr. |
| Kali-carb. | Carb-veg., Phos. |
| Lachesis. | Ars., Calc., Carb-veg., Hep., Lyc., Nitr-ac. |
| Lactic-acid. | Psor. |
| Lycopodium. | Iod., Lach., Puls., Sulph. |
| Magnesia-carb. | Cham. |
| Mercurius. | Aur., Bad., Hep. |
| Mezereum. | Merc. |
| Natrum-mur. | Apis, Arg-n., Sep. |
| Nitric-acid. | Ars., Arum-tryph., Calad., Calc., Lyc. |
| Nux-vomica. | Con., Phos., Sep., Sulph. |
| Opium. | Plb. |
| Palladium. | Plat. |
| Phosphorus. | Ars., Cepa, Kali-c., Sil. |
| Podophyllum. | Calc., Natr-mur., Sulph. |
| Psorinum. | Sulph. |
| Pulsatilla. | Lyc., Sil., Stann., Sulph-ac., Sulph. |
| Rheum. | Magn-c. |
| Rhus-tox. | Bry., Calc., Caust., Sulph. |

| | |
|---|---|
| Ruta. | Calc-phos. |
| Sabadilla. | Sep. |
| Sarsaparilla. | Merc., Sep. |
| Scilla. | Ant-c. |
| Sepia. | Natr-mur., Psor., Sulph. |
| Secale. | Ars., Thuj. |
| Silicea. | Fluor-ac., Thuj. |
| Spongia. | Hepar. |
| Stannum. | Puls. |
| Staphysagria. | Coloc., Caust. |
| Sulphuric-acid. | Puls. |
| Sulphur. | Acon., Aloe., Ars., Bad., Calc., Puls., Pyrogen. |
| Thuja. | Sabin., Sil. |

C. L. Olds.

